# Hepatitis C Worldwide and in Brazil: Silent Epidemic—Data on Disease including Incidence, Transmission, Prevention, and Treatment

**DOI:** 10.1155/2014/827849

**Published:** 2014-06-10

**Authors:** Iara Fabricia Kretzer, Andrea do Livramento, Joel da Cunha, Sabrina Gonçalves, Iraci Tosin, Celso Spada, Aricio Treitinger

**Affiliations:** ^1^Clinical Analysis Department, Health Sciences Center, Federal University of Santa Catarina, 88010-970 Florianópolis, SC, Brazil; ^2^Laboratory of Genetics and Molecular Hematology, University of São Paulo Medical School, HCFMUSP, 05403-010 São Paulo, SP, Brazil; ^3^Department of Microbiology, Immunology and Parasitology, Centre of Biological Sciences, Federal University of Santa Catarina, 88040-900 Florianópolis, SC, Brazil

## Abstract

Hepatitis C virus (HCV) is endemic worldwide and according to the World Health Organization (WHO), there are about 150 million chronic carriers worldwide. The infection is a leading cause of liver diseases like cirrhosis and hepatocellular carcinoma (HCC); thus, HCV infection constitutes a critical public health problem. There are increasing efforts worldwide in order to reduce the global impact of hepatitis C through the implementation of programmatic actions that may increase the awareness of viral hepatitis and also improve surveillance, prevention, and treatment. In Brazil, about 1,5 million people have been chronically infected with HCV. The country has a vast territory with uneven population density, and hepatitis C incidence rates are variable with the majority of cases concentrated in the most populated areas. Currently, the main priorities of Brazilian Ministry of Health's strategies for viral hepatitis management include the prevention and early diagnosis of viral hepatitis infections; strengthening of the healthcare network and lines of treatment for sexually transmitted diseases, viral hepatitis, and AIDS; improvement and development of surveillance, information, and research; and promotion of universal access to medication. This review aims to summarize the available data on hepatitis C epidemiology and current status of efforts in prevention and infection control around the world and in Brazil.

## 1. Introduction

Hepatitis C, a liver disease caused by the hepatitis C virus (HCV), can array in severity from a mild illness, lasting a few weeks, to a serious condition that can lead to chronic liver disease, end-stage cirrhosis, and liver cancer. Worldwide, it is estimated that 350,000 people die every year from complications of hepatitis C disease [1].

The hepatitis C virus is transmitted through contact with infectious blood. There are defined risk factors for hepatitis C virus infection, such as transfusions of HCV-infected blood, contaminated injections during medical procedures, and injection drug use. However, the risk for HCV transmission by sexual or interfamilial contact is not well established [1–3].

According to the World Health Organization (WHO), viral hepatitis is not being addressed seriously, since the early stages of the disease are silent and because of the insidious way in which chronic liver disease is caused. Many HCV-infected patients are unaware of their disease, and the number of people who have been infected with this virus might be underestimated. Therefore, morbidity and mortality related to hepatitis C are likely to increase over the next years [2]. In this review the aim is to present data regarding incidence, transmission, prevention, and treatment of hepatitis C around the world and in Brazil.

## 2. Worldwide Epidemiology of HCV

HCV is endemic worldwide and there is a large degree of geographic variability in its distribution [7]. According to WHO, there are about 150 million chronic carriers worldwide and 3 to 4 million people are infected annually [1]. Worldwide, the prevalence of HCV infection is estimated to be highest in Middle East and also in Africa and lowest in much of Europe and the Americas [8].

Table 1 summarizes the prevalence of HCV infection in different regions of the world. It is shown that, in Africa, the greatest prevalence occurs in Central Africa, followed by the West Africa, Sub-Saharan Africa, and by the lower prevalence estimate found in Southern and East Africa [9, 10].

In the region of the Americas, North America countries such as Canada and the United States present low HCV prevalence rates [11–13]. Regarding Latin America, the presence of poorly designed studies and the regional diversity of social, economic, cultural, and environmental factors are illustrated in heterogeneous HCV prevalence data that can occasionally be dissimilar within the same country [14]. Between 7 and 9 million adults are estimated to be anti-HCV positive in Latin America and the Caribbean countries, and as those individuals were exposed to HCV, they could contract chronic infection [2, 15].

An estimated 17 million people in the Eastern Mediterranean region suffer from chronic HCV infection and approximately 800.000 people are infected with HCV annually [2], and Egypt is believed to have one of the highest rates of hepatitis C in the world [16, 17]. In this case, the main reason for the high prevalence rates that reach 20–30% in young male adults was the mass programmes for the treatment of endemic schistosomiasis that frequently used unsterilized needles [18, 19].

In the European region, approximately nine million people are chronically infected with HCV [2] and according to European Centre for Disease Prevention and Control (ECDC), HCV infection is the most common type of viral hepatitis reported in the European Union (EU) and European Economic Area/European Free Trade Association (EEA/EFTA) countries. In 2009, the overall HCV infection rate of 8.19 per 100,000 population was reported for 26 EU and EEA/EFTA Member States [20]. In 2011, Cornberg et al. [21] reviewed the prevalence of HCV in several countries and the lowest HCV prevalence estimates were from northern European countries, whilst the highest prevalence estimates were from Romania and rural areas in Greece and Italy, as well as portions of Russia.

Chronic HCV infection occurs in approximately 30 million people in the Southeast Asia region [2]. In India the majority of the prevalence studies are based on blood banks data and although several studies estimate a prevalence below 2% [22], two studies evaluating HCV prevalence specifically in commercial blood donors revealed astonishing rates of 55.3% and 87.3% [22–24]. Regarding the Western Pacific region, the large geographical and population distribution profile results in a considerable variability in HCV prevalence [16]. Although China's estimated HCV prevalence is under 2% [16], professional blood donors from Hubei Province and from Inner Mongolia Autonomous Region presented much higher rates of 30.1% and 31.9%, respectively [25, 26]. According to Sievert et al. [16], China has more HCV-infected persons than all of Europe or the Americas.

### 2.1. Brazilian Epidemiology

For epidemiological surveillance in Brazil, the Ministry of Health defines the confirmed cases of hepatitis C as those in which the individual meets the conditions of suspected case and presents positive anti-HCV and detectable HCV-RNA. Therefore, the total number of confirmed cases of hepatitis C in Brazil is 82,041 (Table 2) and 75% of the cases were registered in individuals between the ages of 30 to 59 years old [5].

**Figure 1:**
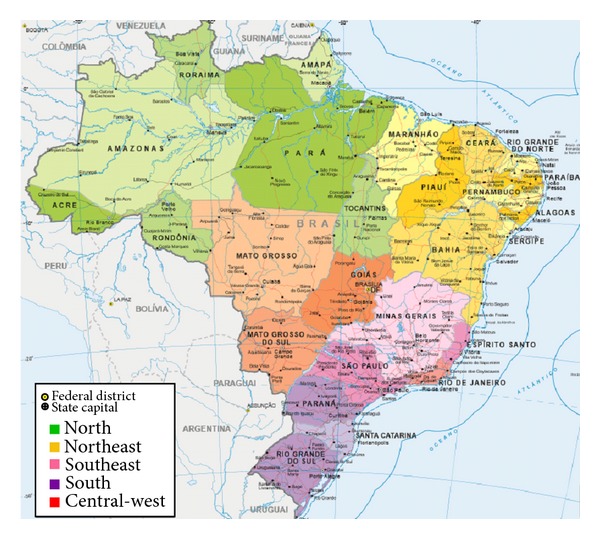
Division of the Brazilian territory into five regional geographic areas [4].

**Figure 2:**
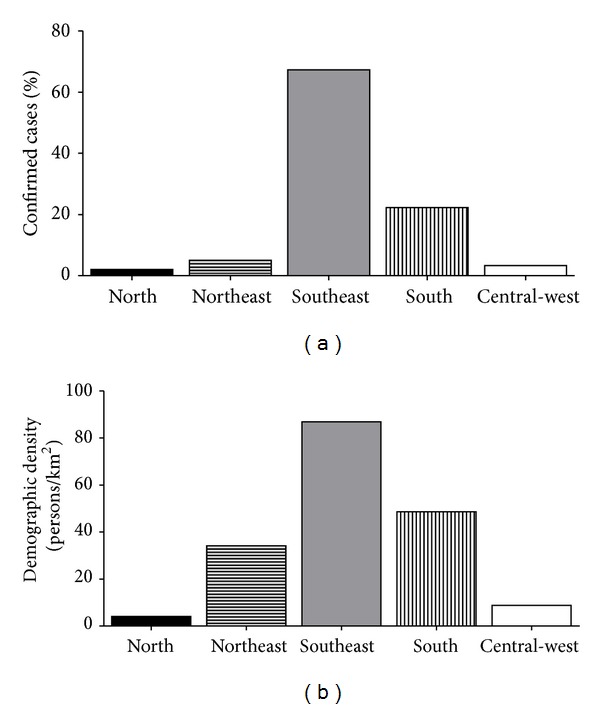
Brazilian regions: (a) distribution of hepatitis C confirmed cases according to the Ministry of Health [5] and (b) population density according to Brazilian Institute of Geography and Statistics [6].

Brazil is divided into five geographic regions according to specific characteristics with regard to physical, human, economic, and cultural aspects (Figure 1). Therefore, the incidence rates of hepatitis C are regionally variable. The majority of cases are concentrated in highly populated areas such as the Southeast (67%) and in the South (22%) (Figure 2) [5, 6]. In those regions, the states of São Paulo, localized in the Southeast Region, and Rio Grande do Sul, localized in the South Region, presented the greatest percentages of confirmed cases until 2011 (85% and 58%, resp.) [5].

Between blood donors, a variant regional distribution of hepatitis C infection was observed in 2002 [30]. Data obtained from blood donor banks revealed that the highest prevalence was reported in the North Region (0.62%), followed by the Northeast (0.55%), the South (0.46%), the Southeast (0.43%), and the West-Center (0.28%) [30, 31].

The Brazilian Ministry of Health launched a nationwide cross-sectional survey to estimate hepatitis infection prevalence in the urban population of all Brazilian state capitals and the Federal District [32, 33]. The survey was performed between years 2005 and 2009 and reported an overall prevalence for anti-HCV marker in all capitals of Brazil of 1.38%. Therefore, those areas were classified as having low endemicity of HCV infection [32–35].

**Figure 3:**
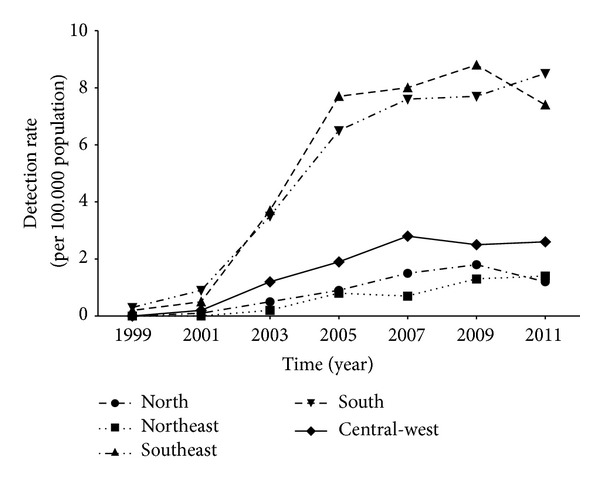
Hepatitis C detection rates in Brazil according to the Ministry of Health [5].

The latest epidemiological report on viral hepatitis produced by the Ministry of Health revealed that, in 2011, the overall hepatitis C detection rate in Brazil was 5 per 100,000 population [5]. As shown in Figure 3, the regions with the most elevated detection rates in 2011 were the South and the Southeast (8.5 and 7.4 per 100,000 population, resp.). The uneven geographical distribution of HCV in Brazil with the higher number of confirmed cases and detection rates in the regions Southeast and South may be explained by the fact that those are wealthier regions which present highly populated (Figure 2(b)) and urbanized areas with better or easier access to the health system [6, 35, 36].

## 3. Prevention and Control of HCV

According to WHO, tailored prevention and control strategies for viral hepatitis are necessary because of the differences in the geographic distribution, transmission, diagnosis, and treatment of hepatitis infections among the world [2]. There is no vaccine available for the prevention of HCV infection; therefore, education, adherence to hygienic standards, and safe injection practices are important strategies in order to prevent HCV transmission [37, 38].

Global efforts to reduce hepatitis C burden could benefit from a focus on primary prevention [39] and WHO states that the risk of HCV infection can be reduced by the avoidance of the use of unsafe blood products; use of illicit drugs and sharing of injection equipment; practice of unprotected sex with HCV-infected people; use of unnecessary and unsafe injections; unsafe sharps waste collection and disposal; sharing of sharp personal items possibly contaminated with infected blood and the use of contaminated equipment for tattoos, piercings, and acupuncture [1]. As for secondary and tertiary prevention, WHO recommendation for infected people includes education and counseling regarding care and treatment of the disease; appropriate early medical management of the disease; regular monitoring for early diagnosis of chronic liver disease; and immunization for the prevention of the coinfection with hepatitis A virus (HAV) and hepatitis B virus (HBV) in order to protect the liver [1].

Surveillance for hepatitis C is important in order to identify HCV-infected people and, therefore, reduce the risks for HCV transmission and development of chronic liver disease [39–42]. In this regard, the Centers for Disease Control (CDC) in the United States recommends the screening for injection drug users; people on long-term dialysis; children born to HCV-infected women; healthcare and public safety workers after accidents resulting in exposure to HCV positive blood; people who received a blood transfusion or organ transplant before the beginning of blood and organs screening in July 1992; and people who received clotting factor concentrates before the development of more advanced methods for manufacturing those products in 1987 [26, 40].

According to Averhoff and colleagues [43], the absence of effective care and treatment programs in many countries may lead to an increase in HCV-associated morbidity and mortality even when control of HCV transmission is realized. Treatment of hepatitis C is usually based on the combined antiviral therapy with interferon alpha and ribavirin and HCV is generally considered to be a curable disease. However, there are some limitations for the success of the treatment including the variation in the response to the treatment from person to person, the patient tolerability to the drugs, and the availability of interferon in many countries [1, 2, 44]. In 2011, two HCV NS3/4a protease inhibitors called boceprevir and telaprevir were approved by the FDA and the European Medicines Agency for treatment of patients with chronic HCV genotype 1 infection [44, 45]. Despite the encouraging results achieved with the triple therapy with pegylated interferon alpha, ribavirin, and the new protease inhibitors [46–49], treatment of chronic HCV is still a challenge since a significant number of patients are not eligible for this therapy [50].

Overall, eradication of HCV around the world is an extremely challenging task that may be possible through a combination of strategies including the education on infection prevention, clinical and targeted community screening, linkage to disease management, and treatment with new therapeutic regimens [51].

### 3.1. Prevention and Control of HCV Infection in Brazil

In Brazil, the department called STD, AIDS, and Viral Hepatitis Department is part of the structure of the Ministry of Health's Surveillance Secretariat and intends to reduce HIV/AIDS and viral hepatitis transmission and to promote patients' quality of life [28, 52]. Among the main priorities actions of this department are the prevention and early diagnosis of viral hepatitis infections and HIV; strengthening of the healthcare network and lines of treatment for STDs, viral hepatitis, and AIDS; improvement and development of surveillance, information, and research; and promotion of universal access to medication and condoms, among others [52].

As recommended by WHO [1], prevention of hepatitis C in Brazil is also focused on primary prevention in order to reduce the risk of disease spreading and on secondary prevention aiming to avoid the progression of the disease in already infected people [53]. According to the Brazilian Ministry of Health's STD, AIDS, and Viral Hepatitis Department, prevention and management strategies should lay emphasis on two generations of people: young people and adults over 45 years old. The main reason regarding the need of special attention to infected adults aging over 45 years is the possible late diagnosis within this population, in which detection of HCV infection may occur only in advanced stages of the disease particularly because their exposure to HCV is related to blood transfusions performed before blood screening in 1993, injection drug use in the past, and performance of hospital procedures such as hemodialysis. As for the young people population, the risk of exposure to HCV is through sharing of drug-use equipment (syringes, needles, and pipes) and frequent practice of tattooing and body piercing without attention to sterilization or use of disposable equipment [54].

In order to promote education to the general population about the risks of hepatitis, modes of transmission, and prevention of those diseases, the Brazilian Ministry of Health promotes several public campaigns aiming to increase the awareness of viral hepatitis and to encourage individuals that could have been exposed to hepatitis C to get free diagnostic tests in Counselling and Testing Centres (CTC) [55–58]. The CTC are public health systems that perform diagnostic tests and promote prevention actions for sexually transmitted diseases (STD), viral hepatitis, and HIV/AIDS such as conduction of private or group counseling sessions, education about prevention of those diseases, and also provision of prevention supplies (male and female condoms for the general population, lubricant gel for sex workers and men who have sex with men, and harm reduction kits for people who use drugs) [59].

As a strategy to improve the access to diagnosis of hepatitis C, the Brazilian Ministry of Health started, in 2011, the distribution of HCV rapid tests for HCV screening to the CTC; the expansion of the network of laboratories that performed molecular biology based tests for diagnosis hepatitis C; and the purchase and distribution of viral load and genotyping tests for hepatitis C [60]. It is important to point out that patients who are diagnosed with HCV infection are referred to specialized services for the proper management and treatment of the disease.

Despite these initiatives, it is worth noting that, in Brazil, about 1,5 million people have been chronically infected with HCV in Brazil and only about 12 thousand people are currently under treatment for hepatitis C, which reflects the large number of individuals without access to diagnosis [54]. Therefore, the expansion of hepatitis C screening is extremely important, since WHO recognizes early diagnosis as a great opportunity for effective medical support, for prevention of further spread, and also for enabling infected persons to take precautions in order to prevent the transmission of hepatitis to others [2].

Among the programmatic actions for the control of hepatitis C in Brazil is the publication by the Ministry of Health of national protocols and clinical practice guidelines on management and treatment of chronic viral hepatitis C [28, 29, 61]. Those guidelines provide the recommendations for clinical and therapeutic approaches regarding management of key situations found in routine care of patients with hepatitis C. Initially, the proposed drugs were interferon alpha, pegylated interferon alpha, and ribavirin [28], and more recently, in 2013, boceprevir and telaprevir were also recommended for the treatment of patients chronically infected with HCV genotype 1 with advanced fibrosis [29, 61]. The current major recommendations for hepatitis C treatment in Brazil according to the Ministry of Health are summarized in Table 3.

A national survey about acute hepatitis C was coordinated by the Brazilian Society of Hepatology between the years 2000 and 2008 [62]. The study revealed concern with respect to therapeutic management of the disease since no standard therapeutic regimen could be identified among all studied services. The various treatment regimens reported in the study included conventional or pegylated interferon, which was administered alone or in combination with ribavirin for 16 to 72 weeks, with a sustained virological response (SRV) rate of 60%. Furthermore, the beginning time of treatment was shown to be longer than that recommended with a median of 24 weeks. Therefore, the avoidance of a late onset of treatment was recommended aiming at the achievement of optimized cure rates and reduction of the possible progression to chronic forms of HCV infection.

Regarding the treatment of chronic hepatitis C, Azevedo and colleagues [63] demonstrated SVR rates of approximately 30% in patients who received either conventional interferon or pegylated interferon in combination with ribavirin. The same SVR rate was shown in another study with patients who received only the conventional interferon and ribavirin [64]. A number of studies with hepatitis C genotype 1 patients treated with pegylated interferon in combination with ribavirin revealed SVR rates ranging from approximately 35 to 52% [65–68].

Most of the studies evaluating the efficacy of the treatment for chronic hepatitis C in Brazil were performed before the recent introduction of boceprevir and telaprevir for the treatment of patients chronically infected with HCV genotype 1 with advanced fibrosis. Therefore, there is expectation for the possibility of better treatment responses with the use of protease inhibitors in those patients. Further studies will be necessary in order to evaluate the impact of those drugs in the treatment of chronic hepatitis C in Brazil, especially because HCV genotype 1 is common in the country [5].

In a study on the cost of hepatitis C treatment for the Brazilian Health System, Blatt and colleagues [69] have demonstrated that the treatment of HCV infection in Brazil is considered expensive taking into account the cost of antiviral drugs and the demands on medical resources. It was estimated that the total direct costs (per patient) of hepatitis C treatment with interferon plus ribavirin, with pegylated interferon alpha-2a plus ribavirin, and with pegylated interferon alpha-2b plus ribavirin were US $982.25, US $10,658.08, and US $12,597.63, respectively. The study revealed that the most expensive element of the total cost of treatment is the antiviral drugs, which are responsible for more than 40% of the medical costs of interferon plus ribavirin therapy and more than 88% of pegylated interferon plus ribavirin therapy.

Since there is no vaccine available in order to prevent HCV infection, the need for improvement of prevention strategies is particularly important regarding hepatitis C control. The estimated absolute numbers of infected individuals shown by the national seroepidemiological survey of hepatitis A, B, and C infection indicated the burden of hepatitis C in the near future [35]. In addition, it is also noteworthy that the risk factors identified within the survey explained less than 50% of the identified HCV infection cases, a fact that may limit the effective implementation of prevention strategies [35]. The study also pointed out the necessity of improving the strategies for reducing transmission among drug users and nosocomial infection in Brazil. In fact, the rigorous implementation of preventive measures in order to minimize nosocomial transmission in Brazil was also recommended in a national survey on acute hepatitis C that was coordinated by the Brazilian Society of Hepatology [62].

Prevalence studies may be useful tools not only to verify the impact of the existing hepatitis prevention strategies on the disease epidemiology, but also to create novel disease control strategies. However, Brazilian hepatitis prevalence studies usually present characteristics that may limit their results as of representative value for the whole country: most studies are conducted in restricted areas and not nationwide [70–74] or include only urban areas of the states' capitals such as those observed with the Brazilian National Survey of Viral Hepatitis [32, 34, 35]. Furthermore, most studies are conducted in specific populations, such as blood donors, and not as general population based studies [74–76]. Therefore, further studies should be encouraged so that the real impact of the implemented prevention strategies could be established for the whole country.

## 4. Final Disclosures

The need for improvement of prevention activities is particularly important regarding hepatitis C, since there is no vaccine available in order to prevent HCV infection. In this regard, comprehensive testing strategies must ensure the identification of infected individuals who are at increased risk for spreading hepatitis C virus.

In addition, public-health measures worldwide should focus on approaches that may increase the awareness of hepatitis C and also expand the capacity of health systems with respect to surveillance, prevention, treatment, and implementation of effective programmatic actions to control the disease. In this context, implementation of standard precautions in health-care facilities to avoid nosocomial infection and effective public education seem to be the key tools in order to reduce the global impact of HCV infection.

In Brazil, the number of hepatitis C confirmed cases slightly decreased since 2010 (Table 2), probably as a result of the government's efforts for the improvement of prevention and control of viral hepatitis. Thus, advances in surveillance, diagnosis methods, and disease control programs may have facilitated the identification of cases, treatment of infected patients, and decreasing transmissibility of the virus. On the other hand, it is important to point out that, in Brazil, an additional challenge in order to increase control and management of hepatitis C is imposed by the vast territorial dimension and the consequent enormous differences regarding culture, population density, income distribution, and health conditions among the five political regions into which the country is divided. The efficacy of government efforts for HCV infection management is still affected by those geographical and socioeconomic aspects, and overcoming these difficulties is an arduous but crucial task for the success in hepatitis C control.

## Figures and Tables

**Table 1:** Worldwide prevalence of hepatitis C virus.

Region	Prevalence of HCV
Africa [9, 10]	
Sub-Saharan Africa	2.2% (0.1%–13.8%)
Central Africa	6%
West Africa	2.4%
Southern and East Africa	1.6%

Americas [11–13, 15]	
North America	
Canada	0.7%
United States	1.3%
Latin America	
Argentina, Brazil, Mexico, Puerto Rico, Peru, and Venezuela	1.4–2.5%

Asia and Oceania [2, 16, 22–27]	
South Asia	
India	3.4%
Southeast Asia	
Vietnam	2–2.9%
East Asia	
Taiwan	4.4%
China	1–1.9%
Australasia (Australia and New Zealand)	2.7%
Melanesia,Micronesia,and Polynesia regions	2.6%

Eastern Mediterranean [10, 16, 17]	
Egypt	15%
Pakistan	4.9%

Europe [21]	
Central Europe	
Czech Republic, Poland, Romania, and Hungary	≤0.5%
Romania	≥3%
Western Europe	
France, Germany, Greece, Italy, Norway, Portugal, Spain, Sweden, Switzerland, and UK	≤0.5%
Rural areas in Greece and in Italy	≥3%
Eastern Europe	
Russia	≤0.5%
Parts of Russia	≥3%

**Table 2:** Hepatitis C confirmed cases in Brazil according to the Ministry of Health [5].

Region	Number of cases among the years													
	1999	2000	2001	2002	2003	2004	2005	2006	2007	2008	2009	2010	2011	1999– 2011
Brazil	188	309	632	2,031	4,021	7,135	8,572	9,280	9,517	9,936	10,534	10,321	9,565	82,041
North	2	30	19	34	70	68	128	100	226	268	274	230	195	1,644
Northeast	1	0	6	34	106	208	383	426	382	549	671	637	728	4,131
Southeast	110	166	363	1,423	2,791	5,126	6,073	6,600	6,430	6,571	7,095	6,528	5,946	55,222
South	72	111	223	400	910	1,530	1,743	1,924	2,105	2,248	2,143	2,561	2,337	18,307
Central-west	3	2	21	140	144	203	245	230	374	300	351	365	359	2,737

**Table 3:** Recommendations for the treatment of hepatitis C in Brazil according to the Ministry of Health [28, 29].

Hepatitis C Treatment	
Acute	

(i) Conventional interferon (IFN) monotherapy in a daily dose of induction (alpha-2a at a dose of 6 MUI or alpha-2b at a dose of 5 MUI), subcutaneously (SC), in the first 4 weeks followed by 3 MUI three times per week for 20 weeks (completing 24 weeks of treatment); or (ii) conventional IFN-alpha-2a or alpha-2b, 3 MUI, SC, three times per week, associated to oral ribavirin (RBV) 15 mg/kg/day orally for 24 weeks, for those patients at higher risk of intolerance and/or poor treatment adhesion to higher doses of conventional IFN	

Chronic hepatitis genotype 1	

Association of pegylated interferon (PEG-IFN) and RBV for 48 to 72 weeks: PEG-IFN alpha-2a, 180 mcg, SC, 1 time per week associated with oral RBV 15 mg/kg/day; or PEG-IFN alpha-2b, 1.5 mcg/kg, SC, one time per week, associated with oral RBV 15 mg/kg/day	

Chronic hepatitis monoinfected with genotype 1 and with advanced fibrosis^a ^or compensated liver cirrhosis^b^	

(i) Triple therapy with PEG-IFN alpha, RBV, and telaprevir: oral telaprevir 750 mg, taken 3 times a day (8 hours apart), administered with PEG-IFN alpha and RBV for 4 or 12 weeks (depending on viral response), followed by a response-guided regimen of either 12 or 36 additional weeks of PEG-IFN alpha and RBV (depending on viral response); oral telaprevir 750 mg, taken 3 times a day (8 hours apart), administered with PEG-IFN alpha and RBV. Therapy initiated with double therapy with PEG-IFN alpha and RBV for 4 weeks, and then addition of telaprevir in a triple therapy for 4 or 12 weeks (depending on viral response), followed by a response-guided regimen of either 12 or 32 additional weeks of double therapy with PEG-IFN alpha and RBV (depending on viral response). (ii) Triple therapy with PEG-IFN alpha, RBV, and boceprevir^c^: oral boceprevir 800 mg administered orally three times daily (8 hours apart), administered with PEG-IFN alpha and RBV. Therapy initiated with double therapy with PEG-IFN alpha and RBV for 4 weeks and then addition of boceprevir in response-guided regimen of either 8, 20, or 44 additional weeks of triple therapy	

Chronic hepatitis genotypes 2 and 3 in the absence of predictors of low sustained virologic response (SVR)^d,e^	

Combination of conventional IFN and RBV for 24 weeks: conventional INF alpha-2a or alpha-2b, 3 MUI, SC, 3 times per week, associated with oral RBV 15 mg/kg/day	

Chronic hepatitis genotypes 2 and 3 in the existence of predictors of low SVR^e^	

Combination of PEG-IFN and RBV for 24 to 48 weeks: PEG-IFN alpha-2a or PEG-IFN alpha-2b, once a week, SC, associated with oral RBV 15 mg/kg/day	

Chronic hepatitis genotypes 4 and 5	

Association of PEG-IFN alpha and RBV for 48 to 72 weeks: PEG-IFN alpha-2a, 180 mcg, SC, 1 time per week associated with oral RBV 15 mg/kg/day; or PEG-IFN alpha-2b, 1.5 mcg/kg, SC, one time per week, associated with oral RBV 15 mg/kg/day	

^a^Metavir F3 and F4; or patients with evidence of portal hypertension by endoscopy or imaging tests.

^b^Patients with compensated liver disease (Child-Pugh score ≤ 6; class A), with no history of previous decompensation.

^c^May be considered for patients with advanced fibrosis (Metavir F3 and F4/cirrhosis) according to criteria for individualization of treatment that contraindicates the use of telaprevir for 12 weeks.

^d^Patients who have predictors of low response to the treatment with conventional INF should receive treatment with PEG-IFN.

^e^Predictors of low response to the treatment with conventional INF: METAVIR score ≥ F3; and/or clinical manifestations of liver cirrhosis; and/or viral load higher than 600,000 UI/mL.
